# Genotoxicity of Superparamagnetic Iron Oxide Nanoparticles in Granulosa Cells

**DOI:** 10.3390/ijms161125960

**Published:** 2015-11-03

**Authors:** Marina Pöttler, Andreas Staicu, Jan Zaloga, Harald Unterweger, Bianca Weigel, Eveline Schreiber, Simone Hofmann, Irmi Wiest, Udo Jeschke, Christoph Alexiou, Christina Janko

**Affiliations:** 1 Department of Otorhinolaryngology, Head and Neck Surgery, Section for Experimental Oncology and Nanomedicine (SEON), Else Kröner-Fresenius-Stiftung Professorship, University Hospital Erlangen, Glückstraße 10a, Erlangen 91054, Germany; andreas.staicu@uk-erlangen.de (A.S.); jan.zaloga@uk-erlangen.de (J.Z.); harald.unterweger@uk-erlangen.de (H.U.); bianca.weigel@uk-erlangen.de (B.W.); eveline.schreiber@uk-erlangen.de (E.S.); christoph.alexiou@uk-erlangen.de (C.A.); christina.janko@uk-erlangen.de (C.J.); 2 Department of Obstetrics and Gynecology, Ludwig Maximilians University of Munich, Maistraße 11, Munich 80337, Germany; simone.hofmann@med.uni-muenchen.de (S.H.); irmgard.wiest@med.uni-muenchen.de (I.W.); udo.jeschke@med.uni-muenchen.de (U.J.)

**Keywords:** superparamagnetic iron oxide nanoparticles, protein corona, cancer therapy and diagnosis, reproductive health, granulosa cells

## Abstract

Nanoparticles that are aimed at targeting cancer cells, but sparing healthy tissue provide an attractive platform of implementation for hyperthermia or as carriers of chemotherapeutics. According to the literature, diverse effects of nanoparticles relating to mammalian reproductive tissue are described. To address the impact of nanoparticles on cyto- and genotoxicity concerning the reproductive system, we examined the effect of superparamagnetic iron oxide nanoparticles (SPIONs) on granulosa cells, which are very important for ovarian function and female fertility. Human granulosa cells (HLG-5) were treated with SPIONs, either coated with lauric acid (SEONLA) only, or additionally with a protein corona of bovine serum albumin (BSA; SEON^LA-BSA^), or with dextran (SEON^DEX^). Both micronuclei testing and the detection of γH2A.X revealed no genotoxic effects of SEON^LA-BSA^, SEON^DEX^ or SEON^LA^. Thus, it was demonstrated that different coatings of SPIONs improve biocompatibility, especially in terms of genotoxicity towards cells of the reproductive system.

## 1. Introduction

Superparamagnetic iron oxide nanoparticles (SPIONs) have been widely investigated for many years now. Due to their exceptional magnetic, electronic and optical properties, they have turned out to be promising candidates for research and future use in an industrial or clinical setting. Especially for medical and scientific applications ranging from *in vitro* diagnostic tests, *in vivo* imaging, targeted drug delivery and tissue regeneration, SPIONs are capable candidates.

In particular, SPION-based contrast enhancement in magnetic resonance imaging (MRI) [1], magnetic hyperthermia treatment [2] and magnetic drug targeting (MDT) [3,4] are of special interest in the therapy and diagnosis of cancer and other diseases [3,5]. Their incorporation into therapeutic drugs and their parallel use in imaging processes enables SPIONs to become “theranostic” agents. Additionally, the use of SPIONs in magnetic tissue engineering is a new concept in biomedicine [6].

SPIONs usually consist of iron oxide cores measuring 5–20 nm in diameter made of magnetite (Fe_3_O_4_) and its oxidized form maghemite (γ-Fe_2_O_3_). To increase their colloidal stability and biocompatibility, these iron oxide cores are coated with, e.g., long-chain fatty acids [7] or biocompatible polymers as chitosan or dextran [8]. Commercially accessible contrast agents like Sinerem, Resovist, Supravist and Ferridex have a surface coating of dextran or carboxydextran [9]. The formation of a surrounding protein corona also contributes to the stabilization and biocompatibility of iron oxide nanoparticles [10].

Because of the highly catalytic properties of nanoparticle surfaces [11,12], their coating may work as a barrier and could reduce their toxic potential. Especially for iron oxide nanoparticles, Fenton-like reactions caused by released iron ions [13] or on the nanoparticle surface have been under discussion as triggering toxic effects [14]. Here, hydroxyl radicals are generated which are highly reactive and react with almost all cellular macromolecules such as lipids, proteins, and carbohydrates. Since nanotoxicity has been identified as being a tiered process starting with oxidative stress, the oxidation of cellular components may finally result in cell death [12]. It is an important fact that oxidative stress has also been identified as causing DNA damage such as abasic DNA sites, oxidized bases along with single and double strand DNA breaks [15].

For the future translation of SPIONs from bench to bedside, it is crucial to evaluate their biocompatibility and exclude potential toxic effects. Only few studies have focused to date on the effect of nanoparticles on reproductive cells. Since iron oxide nanoparticles have previously been shown to cross the placenta and accumulate in the fetus [16], applied medical nanoparticles must be absolutely biocompatible and safe. Here, granulosa cells are used as a model system for female reproductive tissue. These cells play a key role in sustaining ovarian function, health and female fertility and, are thus closely associated with the development of the female gamete. In this study, we compare the effect of SPIONs which were coated with different surface moieties. The first two systems, SEON^LA^ and SEON^LA-BSA^ derive from the same coprecipitation synthesis where the particles are stabilized *in situ* by a double layer of lauric acid [17]. The difference is that SEON^LA-BSA^ is additionally coated with a BSA shell, which greatly improves colloidal stability, influences biocompatibility and enhances its capacity for drug loading. In a recent, detailed study, we comprehensively characterized the properties of these two systems [10]. The third system is synthesized also by coprecipitation, but a different surface coating strategy was chosen: SEON^DEX^ particles are directly precipitated in dextran containing iron solution. This enables narrow core size distribution and high colloidal stability by steric stabilization. These particles have also been comprehensively characterized earlier [18]. As an important aspect, we demonstrated that the appropriate coating of iron oxide nanoparticles ensures their biocompatibility.

## 2. Results and Discussion

### 2.1. Uptake of Iron Oxide Nanoparticles by Granulosa Cells

**Figure 1:**
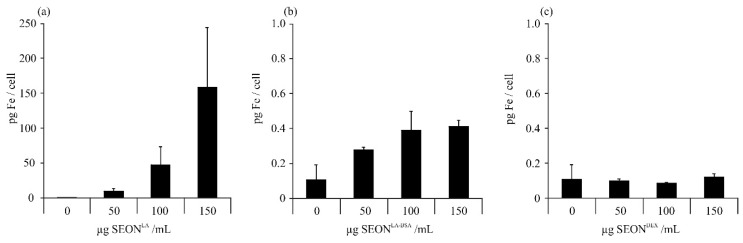
Uptake of SPIONs by HLG-5 cells. Cells were incubated with 50, 100 or 150 μg/mL of (**a**) SEON^LA^; (**b**) SEON^LA-BSA^; or (**c**) SEON^DEX^ for 48 h. The cellular iron content was analyzed from cell lysates by microwave plasma atomic emission spectroscopy (MP-AES). The mean values of *n* = 3 with standard deviations are shown.

Nanoparticle-induced toxicity is highly correlated with cellular uptake. Therefore, we measured the cellular iron content on equal terms as in toxicity tests. Granulosa cells were incubated for 48 h with three different superparamagnetic iron oxide nanoparticles: SEON^LA^ (coated with lauric acid only), SEON^LA-^^BSA^ (coated with lauric acid and albumin) and SEON^DEX^ (coated with dextran). After incubation, the cells were washed and the amount of iron was subsequently analyzed from cell lysates by microwave plasma atomic emission spectroscopy (MP-AES). Evaluation of cellular iron content indicated that SEON^LA^ were effectively taken up by cells, whereas SEON^LA-BSA^ were only weakly taken up and SEON^DEX^ not at all (Figure 1). These results are in concordance with previous investigations on uptake of SEON nanoparticles by primary human umbilical vein endothelial cells (HUVEC) and by T-cells (Jurkat) [19,20]. Other groups also showed that cellular uptake efficiency of iron oxide nanoparticles is dependent on surface coating and the protein corona [21,22]. To sum up, the presence of a pre-formed albumin protein corona (SEON^LA-BSA^) reduces cellular uptake of the SEON particles remarkably compared to particles stabilized only by a lauric acid layer (SEON^LA^).

### 2.2. Viability of Granulosa Cells after Incubation with SPION

**Figure 2:**
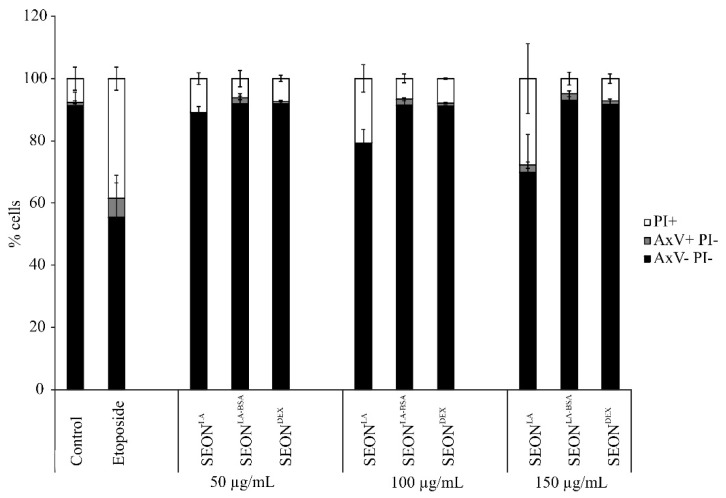
Cell death induction in HLG-5 cells. Cells were incubated with 50, 100, 150 μg/mL SPIONs for 48 h. Etoposide-treated cells served as positive control; mock treated cells served as negative control. Cell viability was determined by AnnexinV-Fitc/propidium iodide (AxV/PI) staining. AxV-/PI- cells are considered viable, AxV+/PI- are apoptotic, and PI+ cells are necrotic. The mean values of *n* = 3 with standard deviations are shown.

Viability of HLG-5 granulosa cells was determined using flow cytometry. The cells were stained for phosphatidylserine exposure using Annexin V-Fitc (AxV) and plasma membrane integrity using propidium iodide (PI). AxV/PI data were confirmed by staining for mitochondrial membrane potential using DiIC_1_(5) (data not shown) according to Munoz *et al.* [23]. AxV/PI staining showed that SEON^LA-BSA^ and SEON^DEX^ did not induce any cytotoxicity up to a tested concentration of 150 μg/mL, whereas SEON^LA^ induced cell death starting at 100 μg/mL (Figure 2). The rate of necrotic cells increased in a dose-dependent manner in SEON^LA^-treated cells.

As granulosa cells do not only protect the oocyte physically, but are furthermore very important for development, toxic effects of nanoparticles on these cells might be accompanied by reduced fertility or congenital defects. Although only few studies have focused on the effect of nanoparticles on reproductive cells to date, it has been demonstrated so far that magnetic nanoparticles do not affect functionality [24], whereas ZnO and TiO_2_ nanoparticles may have toxic effects on male gametes depending on their concentration and composition, and can affect sperm cell functionality [25]. Concerning female gametes, quantum dots have proved to be cytotoxic, consequently negatively influencing oocyte maturation and fertilization [26].

### 2.3. Micronuclei Formation in Granulosa Cells after Incubation with SPION

Micronuclei tests are used in toxicological screening to identify genotoxic substances according to OECD guidelines. Micronuclei are small cytoplasmic bodies formed in anaphase of mitosis or meiosis. They contain pieces of chromosomes, resulting in a lack of DNA information in one daughter cell. In microscopy, they can be recognized as small nuclei separate from the main nucleus and are enclosed in their own nuclear membrane. An augmented frequency of micronuclei serves as a biomarker for genotoxicity [27,28].

**Figure 3:**
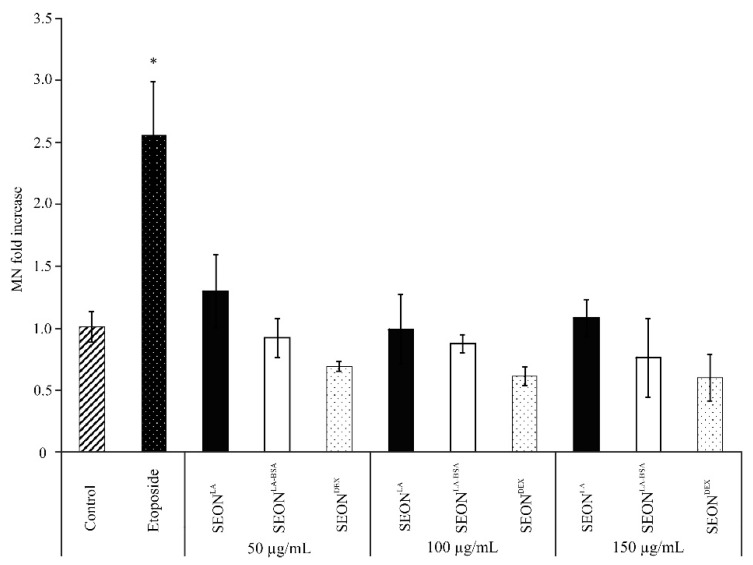
Flow cytometry of micronuclei in HLG-5 cells. Cells were treated with 50, 100 or 150 μg/mL iron oxide nanoparticles. Etoposide-treated cells served as positive control; mock treated cells served as negative control. After 48 h flow cytometry analysis using ethidium monoazide (EMA)/SYTOX green staining revealed no increase in micronuclei induction for SEON^LA-BSA^ and SEON^DEX^ compared to the control (* *p* < 0.01, *n* = 3).

**Figure 4:**
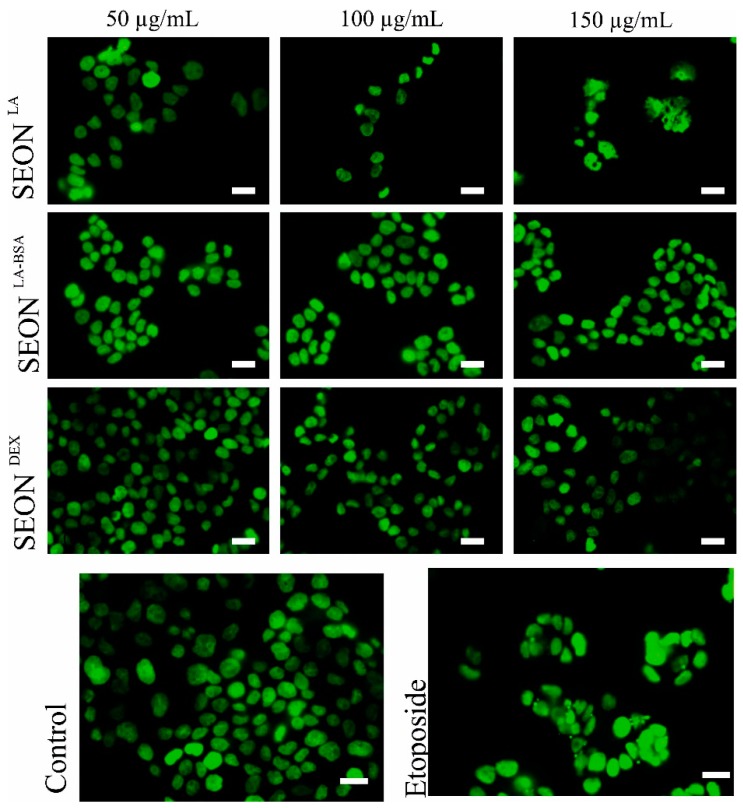
Fluorescence microscopy of micronuclei in HLG-5 cells. HLG-5 cells were treated for 48 h with 50, 100 and 150 μg/mL SEON^LA^, SEON^LA-BSA^ and SEON^DEX^, then DNA was stained with SYTOX green; SEON^LA-BSA^ and SEON^DEX^ showed no effect on cell morphology, whereas SEON^LA^-treated cells appeared unhealthy compared to control. Many micronuclei can be recognized in etoposide-treated cells; (scale bars = 20 μm, representative pictures are displayed; *n* = 3).

Because of their small size some nanoparticles can easily penetrate through membranes directly to the nucleus. Here, they can interact with the DNA and thus being a potential genotoxic hazard [29]. Different concentrations (as indicated) of iron oxide nanoparticles SEON^LA-BSA^, SEON^DEX^ and SEON^LA^ were incubated with granulosa cells and analyzed for micronuclei formation after 48 h (Figure 3 and Figure 4) and 72 h (data not shown) using flow cytometry and fluorescence microscopy. Flow cytometry analysis revealed no remarkable difference in the micronuclei number of SEON^DEX^ and SEON^LA-BSA^ treated cells as compared to the untreated control (Figure 3). Whereas, for SEON^LA^ induction of micronuclei was 0.12-fold higher on average compared to control. This was confirmed via fluorescence microscopy (Figure 4). Vinblastine, which causes M phase specific cell cycle arrest by disrupting microtubule association (not shown), and the topoisomerase IIα inhibitor etoposide were used as positive controls to induce micronuclei formation. So far, it is not clear whether cytotoxicity caused by high concentrations of SEON^LA^ is a secondary effect of DNA damage and will have to be further investigated.

### 2.4. DNA Damage in Granulosa Cells after Incubation with SPION

Since micronuclei formation can be caused by DNA double strand breaks, this was evaluated by detection of phosphorylated H2A.X (Ser139) and ATM (Ser1981). The topoisomerase IIα inhibitor etoposide is a very effective inductor of DNA double strand breaks, and brings cells into G_2_/M phase cell cycle arrest [30]. Following DNA double strand breaks, cell cycle checkpoint arrest and DNA repair requires phosphorylation of histone H2A.X at serine 139 by kinases such as ataxia telangiectasia mutated (ATM). Therefore, the phosphorylation of H2A.X (γ-H2A.X) and ATM (pATM) is an important indicator of DNA damage [31].

**Figure 5:**
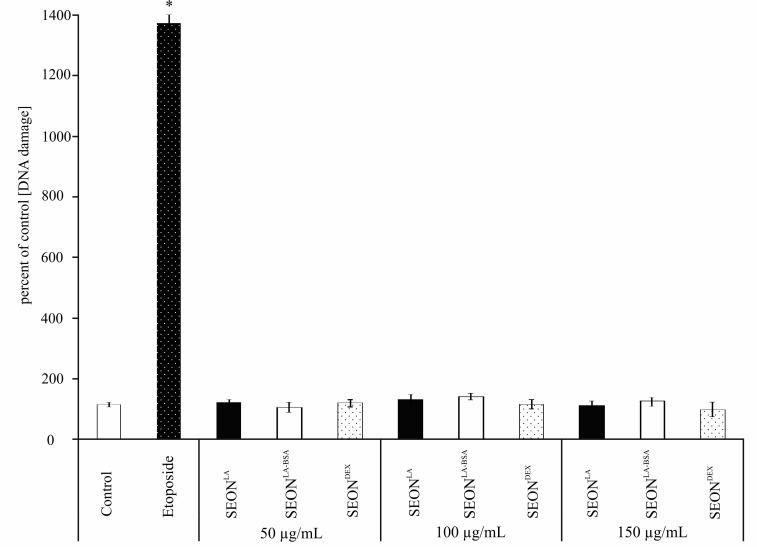
DNA damage (phosphorylation of H.2AX and ATM) in HLG-5 cells. Cells were incubated for 48 h with 50, 100 and 150 μg/mL SEON^LA^, SEON^LA-BSA^ and SEON^DEX^. Mock treated cells served as negative controls; etoposide-treated cells served as positive controls (* *p* < 0.01, *n* = 3).

Treatment of granulosa cells for 48 h with SEON^LA^ , SEON^LA-BSA^ and SEON^DEX^ did not lead to phosphorylation of H2A.X or ATM compared to control cells, here summarized as “DNA damage” (Figure 5). This was also verified by western blot analysis (data not shown). 

Different sources of DNA damage (exogen, endogen, mechanical) can cause a variety of DNA lesions and can thus induce various cellular reactions including cell cycle arrest, apoptosis and notably, DNA repair. DNA double strand breaks are supposed to be the furthermost disastrous forms of DNA destruction, conceding genomic stability. Therefore, it is very important to ensure nanoparticles safety upon DNA damage [32].

## 3. Experimental Section

### 3.1. Nanoparticles

All iron oxide nanoparticles used were comprehensively physico-chemically characterized previously by Zaloga *et al.*, and Unterweger *et al.* [10,18]. Briefly, superparamagnetic iron oxide nanoparticles (SPIONs) were synthetized by co-precipitation in aqueous media (core size 7.64 ± 1.6 nm) and subsequent *in-situ* coating with lauric acid (LA), resulting in SEON^LA^ to form a stable colloid. They were then additionally coated with bovine serum albumin (BSA) by dilution in excess protein solution and following removal of the unbound protein by ultrafiltration, resulting in SEON^LA-BSA^ [10,33]. Upon formation of a BSA protein corona the ζ potential decreased drastically, indicating the high stability of aqueous dispersions of SEON^LA-BSA^. As expected, the surface charge of the SEON^LA-BSA^ particles was pH dependent, with the point of zero charge being just below pH 5 which is very consistent with the isoelectric point of BSA [10].

For the synthesis of SEON^DEX^, SPIONs were covered with dextran, the suspension was ultrafiltrated, and particle-bound dextran finally crosslinked [18]. In SEON^DEX^ particles (core size 4.3 ± 0.9 nm) the dextran content during coprecipitation had an influence on the ζ potential. Formation of a stable colloid was first achieved with 2.0 g of dextran with a ζ potential of −2.0 ± 0.6 nm. SEON^DEX^ show an agglomeration of roundish magnetite particles embedded in a polymer matrix.

Synthesized SPIONs have a spherical morphology; Table 1 provides a summary of the basic physico-chemical nanoparticle characteristics.

**Table 1:** Basic physico-chemical properties of SEON^LA^, SEON^LA-BSA^ and SEON^DEX^ [10,18].

Physico-Chemical Properties of Nanoparticles	SEON^LA^	SEON^LA-BSA^	SEON^DEX^
Core diameter (TEM) (nm) in H_2_O	7.64 ± 1.6	7.64 ± 1.6	4.3 ± 0.9
Hydrodynamic diameter (DLS) (nm) in RPMI	46.92 ± 0.1	61.7 ± 1.1	79 ± 1.3
ζ potential (mV) in RPMI	−15.5 ± 0.8	−12.9 ± 0.55	−2.0 ± 0.6
Polydispersity index in RPMI	0.331 ± 0.019	0.346 ± 0.028	0.304 ± 0.031

### 3.2. Cell Culture

Briefly, human luteinized granulosa cells, HLG-5 were collected from infertile women undergoing In Vitro Fertilization (IVF) pre-embryo transfer treatment [34]. These cells duplicate every 48 h and were maintained in DMEM complemented with 10% fetal calf serum (FCS) (both Biochrom, Berlin, Germany) under standard cell culture conditions in a humidified incubator (INCOmed, Memmert, Schwabach, Germany) at 37 °C and 5% CO_2_. The cells were verified to be free of mycoplasma. For the experiments, the cells were grown to a confluence of 75% and passaged twice a week using 0.25% trypsin/0.02% EDTA in PBS (PAN Biotech, Aidenbach, Germany).

### 3.3. Live/Dead Staining Using Flow Cytometry

HLG-5 cells were seeded at a concentration of 2 × 10^5^ cells/ mL in 24 well plates. SEON nanoparticles (50, 100 and 150 μg/mL) were added in 0.5 mL; mock treated cells served as controls. After 24, 48, and 72 h the cells were trypsinized and resuspended in 1 mL DMEM. Fifty μL of cell solution was incubated with 250 μL of freshly prepared staining solution, containing 1 μg/mL Annexin V-Fitc (AxV-Fitc), 1 μg/mL Hoechst 33342 (Hoe), 5.1 μg/mL DiIC_1_(5) (Dil) (all from Life Technologies, Darmstadt, Germany) and, 20 μg/mL propidium iodide ((PI), Sigma Aldrich, Taufkirchen, Germany) in Ringer’s solution (Baxter Healthcare SA, Zurich, Switzerland) for 20 min at 4 °C [23].

### 3.4. Micronuclei Test

For immunofluorescence staining the culture medium was withdrawn. After a washing step with PBS (Sigma-Aldrich, St. Louis, MO, USA) and fixation with 3.7% formaldehyde (AppliChem, Darmstadt, Germay) for 30 min, cells were permeabilized for 10 min with 0.5% Triton X-100 (Sigma-Aldrich Chemie GmbH, Steinheim, Germany). Afterwards cells were incubated 30 min with RNase (10 mg/mL, Sigma-Aldrich). Staining of the nuclei was achieved with SYTOX green^®^ for 20 min (1 μM, Life Technologies, Eugene, OR, USA). Once one step was finished, slides were washed with PBS. Mounting medium was used to mount coverslips on glass slides (Dako North America, Inc., Carpinteria, CA, USA). The examination was made with a fluorescent microscope Axio Observer Z.1 with an ApoTome (Zeiss, Jena, Germany). Counting and recording of the micronuclei was performed as stated by Tolbert *et al.* [35], with some alterations. Micronuclei are defined as: sphere-shaped forms with a diameter of 1/3 to 1/20 of the central nucleus, micronuclei have to be in the same focus as the nucleus, they should be completely disconnected from the main nucleus and appear with a related shape of chromatin. For each sample set, 3000 cells were scored.

For flow cytometry analysis cells were trypsinized and centrifuged at 600 g for 5 min; supernatant was discarded and cells were resuspended via moderate tapping. After adding 200 μL PBS with 2% heat-inactivated fetal bovine serum (FBS), the cells were transferred into tubes containing 100 μL nucleic acid staining solution (125 mg/mL Ethidium monoazide in PBS with 2% FBS); (EMA, Molecular Probes by Life Technologies). The tubes were cooled in an ice box and photoactiviation was performed with a light source (60 W light bulb, Osram, Munich, Germany) for 20 min and with 30 cm distance to the tubes. Following photoactivation, 800 μL of PBS with 2% FBS was supplemented and samples were transferred into 15 mL tubes with 8 mL PBS with 2% FBS. From this point samples were protected from light. After centrifugation at 600 g for 5 min, the supernatant was discharged. The cells were resuspended by moderate tapping. 1 mL lysis solution 1 (deionized water, 0.584 mg/mL NaCl, 1 mg/mL sodium citrate, 0.3 μL/mL IGEPAL (Sigma-Aldrich), 1 mg/mL RNase and 0.2 μM SYTOX green^®^ was added slowly, the tubes were immediately vortexed for 5 s and were set aside for 1 h at room temperature. Then lysis solution 2 (deionized water, 85.6 mg/mL sucrose, 15 mg/mL citric acid, and 0.2 μM SYTOX green^®^) was added quickly, followed by vortexing and kept at room temperature for another 30 min. Tubes were kept at 4 °C until flow cytometric examination [36]. Tests for statistical significance were carried out using the Student’s *t*-test in MS-Excel (Microsoft Corporation, Redmond, WA, USA).

### 3.5. DNA Damage Detection

HLG-5 cells were seeded at a concentration of 2 × 10^5^ cells/ mL in 12 well plates (TPP Techno Plastic Products, Trasadingen, Switzerland). After 24 h, different SEON nanoparticles (50, 100 and 150 μg/mL) or 10 μM positive control (etoposide) were added in 1 mL; mock treated cells served as controls. After 48 h and 72 h (data not shown) DNA double strand breaks were detected using Muse™ Multi-Color DNA Damage Kit (Merck Millipore, Darmstadt, Germany) [37] by staining 1 × 10^5^ cells of each sample with anti-phospho-Histone H2A.X (Ser139) and anti-phospho-ATM (Ser1981) antibodies. Samples were acquired on the Muse™ Cell Analyzer (Merck Millipore). Tests for statistical significance were carried out using the Student’s *t*-test in MS-Excel (Microsoft Corporation).

### 3.6. Flow Cytometry

Flow cytometry was performed via a Gallios cytofluorometer (Beckman Coulter, Pasadena, CA, USA). Electronic compensation was used to eliminate bleed over fluorescence. The data examination was done with Kaluza software, version 1.2 (Beckman Coulter). All flow cytometry experiments were conducted in triplicate, and the results were averaged.

### 3.7. Microwave Plasma–Atomic Emission Spectrometer (MP-AES)

For determination of absolute cellular iron content, 2 × 10^6^ cells were incubated with 150 μg/mL nanoparticles. After 48 h the cells were washed, cell lysates were prepared from 1 × 10^6^ cells and analyzed via Microwave Plasma–Atomic Emission Spectrometer, MP-AES 4200 (Agilent, Santa Clara, CA, USA). The entire iron level was ascertained at an emission wavelength of 371.993 nm. For calibration external standards of iron at concentrations reaching from 0.01 to 2.5 μg/mL were utilized [38].

## 4. Conclusions

Little is known about the effects of nanoparticles on reproductive tissue and reproductively relevant cells. Gametes and the embryo are rather vulnerable and are therefore located in a more protected environment, but nanoparticles are most likely to cross these barriers, depending on composition, size and/or coating [39,40]. As nanoparticles are already being used in clinics or in clinical studies, they will be part of future medical applications, especially in diagnosis and therapy. Hence, it is of greatest significance to ensure the safety also of reproductive tissue. According to their field of application, e.g., contrast agents for diagnosis or as carriers of therapeutics in Magnetic Drug Targeting (MDT), these particles are coated with different materials. As we found no uptake of SEON^DEX^ particularly in granulosa cells, they are considered to be suitable as contrast agents for magnetic resonance imaging (MRI), because they will most likely remain longer within the blood circulation. On the other hand, SEON^LA-BSA^ with very low toxicity and low uptake can be used for MDT in cancer or atherosclerosis therapy. In this study we demonstrated, that coating of iron oxide nanoparticles is essential to ensure biocompatibility. Future studies are urgently needed to guarantee the safe design of nanoparticles, especially for cells within reproductive tissues.

## References

[1] Jin R., Lin B., Li D., Ai H. (2014). Superparamagnetic iron oxide nanoparticles for MR imaging and therapy: Design considerations and clinical applications. Curr. Opin. Pharmacol..

[2] Silva A.C., Oliveira T.R., Mamani J.B., Malheiros S.M., Malavolta L., Pavon L.F., Sibov T.T., Amaro E., Tannus A., Vidoto E.L. (2011). Application of hyperthermia induced by superparamagnetic iron oxide nanoparticles in glioma treatment. Int. J. Nanomed..

[3] Alexiou C., Tietze R., Schreiber E., Jurgons R., Richter H., Trahms L., Rahn H., Odenbach S., Lyer S. (2011). Cancer therapy with drug loaded magnetic nanoparticles—Magnetic drug targeting. J. Magn. Magn. Mater..

[4] Tietze R., Lyer S., Durr S., Struffert T., Engelhorn T., Schwarz M., Eckert E., Goen T., Vasylyev S., Peukert W. (2013). Efficient drug-delivery using magnetic nanoparticles—Biodistribution and therapeutic effects in tumour bearing rabbits. Nanomed. Nanotechnol. Biol. Med..

[5] Cicha I., Lyer S., Alexiou C., Garlichs C.D. (2013). Nanomedicine in diagnostics and therapy of cardiovascular diseases: Beyond atherosclerotic plaque imaging. Nanotechnol. Rev..

[6] Lee E.A., Yim H., Heo J., Kim H., Jung G., Hwang N.S. (2014). Application of magnetic nanoparticle for controlled tissue assembly and tissue engineering. Arch. Pharmacal. Res..

[7] Tietze R., Lyer S., Durr S., Alexiou C. (2012). Nanoparticles for cancer therapy using magnetic forces. Nanomedicine.

[8] Peng M., Li H., Luo Z., Kong J., Wan Y., Zheng L., Zhang Q., Niu H., Vermorken A., van de Ven W. (2015). Dextran-coated superparamagnetic nanoparticles as potential cancer drug carriers *in vivo*. Nanoscale.

[9] Wang Y.X., Hussain S.M., Krestin G.P. (2001). Superparamagnetic iron oxide contrast agents: Physicochemical characteristics and applications in MR imaging. Eur. Radiol..

[10] Zaloga J., Janko C., Nowak J., Matuszak J., Knaup S., Eberbeck D., Tietze R., Unterweger H., Friedrich R.P., Duerr S. (2014). Development of a lauric acid/albumin hybrid iron oxide nanoparticle system with improved biocompatibility. Int. J. Nanomed..

[11] Nel A., Xia T., Madler L., Li N. (2006). Toxic potential of materials at the nanolevel. Science.

[12] Zhang H.Y., Ji Z.X., Xia T., Meng H., Low-Kam C., Liu R., Pokhrel S., Lin S.J., Wang X., Liao Y.P. (2012). Use of Metal Oxide Nanoparticle Band Gap To Develop a Predictive Paradigm for Oxidative Stress and Acute Pulmonary Inflammation. Acs Nano.

[13] Emerit J., Beaumont C., Trivin F. (2001). Iron metabolism, free radicals, and oxidative injury. Biomed. Pharmacother..

[14] Voinov M.A., Sosa Pagan J.O., Morrison E., Smirnova T.I., Smirnov A.I. (2011). Surface-mediated production of hydroxyl radicals as a mechanism of iron oxide nanoparticle biotoxicity. J. Am. Chem. Soc..

[15] Kryston T.B., Georgiev A.B., Pissis P., Georgakilas A.G. (2011). Role of oxidative stress and DNA damage in human carcinogenesis. Mutat. Res..

[16] Di Bona K.R., Xu Y., Ramirez P.A., DeLaine J., Parker C., Bao Y., Rasco J.F. (2014). Surface charge and dosage dependent potential developmental toxicity and biodistribution of iron oxide nanoparticles in pregnant CD-1 mice. Reprod. Toxicol..

[17] Bica D., Vekas L., Avdeev M.V., Marinica O., Socoliuc V., Balasoiu M., Garamus V.M. (2007). Sterically stabilized water based magnetic fluids: Synthesis, structure and properties. J. Magn. Magn. Mater..

[18] Unterweger H., Tietze R., Janko C., Zaloga J., Lyer S., Durr S., Taccardi N., Goudouri O.M., Hoppe A., Eberbeck D. (2014). Development and characterization of magnetic iron oxide nanoparticles with a cisplatin-bearing polymer coating for targeted drug delivery. Int. J. Nanomed..

[19] Friedrich R.P., Janko C., Poettler M., Tripal P., Zaloga J., Cicha I., Durr S., Nowak J., Odenbach S., Slabu I. (2015). Flow cytometry for intracellular SPION quantification: Specificity and sensitivity in comparison with spectroscopic methods. Int. J. Nanomed..

[20] Gong M., Yang H., Zhang S., Yang Y., Zhang D., Qi Y., Zou L. (2015). Superparamagnetic core/shell GoldMag nanoparticles: Size-, concentration- and time-dependent cellular nanotoxicity on human umbilical vein endothelial cells and the suitable conditions for magnetic resonance imaging. J. Nanobiotechnol..

[21] Gaihre B., Hee Lee Y., Khil M.S., Yi H.K., Kim H.Y. (2011). *In-vitro* cytotoxicity and cell uptake study of gelatin-coated magnetic iron oxide nanoparticles. J. Microencapsul..

[22] Landgraf L., Christner C., Storck W., Schick I., Krumbein I., Dahring H., Haedicke K., Heinz-Herrmann K., Teichgraber U., Reichenbach J.R. (2015). A plasma protein corona enhances the biocompatibility of Au@Fe_3_O_4_ Janus particles. Biomaterials.

[23] Munoz L.E., Maueroder C., Chaurio R., Berens C., Herrmann M., Janko C. (2013). Colourful death: Six-parameter classification of cell death by flow cytometry—Dead cells tell tales. Autoimmunity.

[24] Makhluf S.B.D., Qasem R., Rubinstein S., Gedanken A., Breitbart H. (2006). Loading magnetic nanoparticles into sperm cells does not affect their functionality. Langmuir ACS J. Surf. Colloids.

[25] Gopalan R.C., Osman I.F., Amani A., de Matas M., Anderson D. (2009). The effect of zinc oxide and titanium dioxide nanoparticles in the Comet assay with UVA photoactivation of human sperm and lymphocytes. Nanotoxicology.

[26] Hsieh M.S., Shiao N.H., Chan W.H. (2009). Cytotoxic effects of CdSe quantum dots on maturation of mouse oocytes, fertilization, and fetal development. Int. J. Mol. Sci..

[27] Goel S., Bhatia A., Dey P. (2013). Spontaneously occurring micronuclei in infiltrating ductal carcinoma of breast: A potential biomarker for aggressive phenotype detection?. Diagn. Cytopathol..

[28] Kumar V., Rao N.N., Nair N.S. (2000). Micronuclei in oral squamous cell carcinoma. A marker of genotoxic damage. Indian J. Dent. Res..

[29] Geiser M., Rothen-Rutishauser B., Kapp N., Schurch S., Kreyling W., Schulz H., Semmler M., Im Hof V., Heyder J., Gehr P. (2005). Ultrafine particles cross cellular membranes by nonphagocytic mechanisms in lungs and in cultured cells. Environ. Health Perspect..

[30] Schonn I., Hennesen J., Dartsch D.C. (2010). Cellular responses to etoposide: Cell death despite cell cycle arrest and repair of DNA damage. Apoptosis.

[31] Tanaka T., Huang X., Halicka H.D., Zhao H., Traganos F., Albino A.P., Dai W., Darzynkiewicz Z. (2007). Cytometry of ATM activation and histone H2AX phosphorylation to estimate extent of DNA damage induced by exogenous agents. Cytom. A: J. Int. Soc. Anal. Cytol..

[32] Wan R., Mo Y., Feng L., Chien S., Tollerud D.J., Zhang Q. (2012). DNA damage caused by metal nanoparticles: Involvement of oxidative stress and activation of ATM. Chem. Res. Toxicol..

[33] Zaloga J., Stapf M., Nowak J., Pottler M., Friedrich R.P., Tietze R., Lyer S., Lee G., Odenbach S., Hilger I. (2015). Tangential flow ultrafiltration allows purification and concentration of lauric acid-/albumin-coated particles for improved magnetic treatment. Int. J. Mol. Sci..

[34] Pavlik R., Wypior G., Hecht S., Papadopoulos P., Kupka M., Thaler C., Wiest I., Pestka A., Friese K., Jeschke U. (2011). Induction of G protein-coupled estrogen receptor (GPER) and nuclear steroid hormone receptors by gonadotropins in human granulosa cells. Histochem. Cell Biol..

[35] Tolbert P.E., Shy C.M., Allen J.W. (1991). Micronuclei and other nuclear anomalies in buccal smears—A field-test in snuff users. Am. J. Epidemiol..

[36] Bryce S., Avlasevich S., Raja S., Torous D., Bemis J., Dertinger S. (2009). Flow Cytometric *in vitro* Micronucleus Scoring Provides Simultaneous Mode of Action Information. Environ. Mol. Mutagen..

[37] Santos M., Zhang W., Rollins L., Hsu M. (2014). A Quantitative Approach for Interrogation of H2A.X and ATM DNA Damage Signaling Using Rapid Benchtop Flow Cytometry.

[38] Forte G., Alimonti A., Violante N., di Gregorio M., Senofonte O., Petrucci F., Sancesario G., Bocca B. (2005). Calcium, copper, iron, magnesium, silicon and zinc content of hair in Parkinson’s disease. J. Trace Elem. Med. Biol..

[39] Wick P., Malek A., Manser P., Meili D., Maeder-Althaus X., Diener L., Diener P.A., Zisch A., Krug H.F., von Mandach U. (2010). Barrier capacity of human placenta for nanosized materials. Environ. Health Perspect..

[40] Myllynen P.K., Loughran M.J., Howard C.V., Sormunen R., Walsh A.A., Vahakangas K.H. (2008). Kinetics of gold nanoparticles in the human placenta. Reprod. Toxicol..

